# Recover of Soil Microbial Community Functions in Beech and Turkey Oak Forests After Coppicing Interventions

**DOI:** 10.1007/s00248-024-02402-2

**Published:** 2024-06-28

**Authors:** Enrica Picariello, Flavia De Nicola

**Affiliations:** Department of Sciences and Technologies, University of Sannio, 82100 Benevento, Italy

**Keywords:** Coppice and high forest, Microbial functional diversity, Enzyme activities, Soil organic C

## Abstract

Forest management influences the occurrence of tree species, the organic matter input to the soil decomposer system, and hence, it can alter soil microbial community and key ecosystem functions it performs. In this study, we compared the potential effect of different forest management, coppice and high forest, on soil microbial functional diversity, enzyme activities and chemical-physical soil properties in two forests, turkey oak and beech, during summer and autumn. We hypothesized that coppicing influences soil microbial functional diversity with an overall decrease. Contrary to our hypothesis, in summer, the functional diversity of soil microbial community was higher in both coppice forests, suggesting a resilience response of the microbial communities in the soil after tree cutting, which occurred 15–20 years ago. In beech forest under coppice management, a higher content of soil organic matter (but also of soil recalcitrant and stable organic carbon) compared to high forest can explain the higher soil microbial functional diversity and metabolic activity. In turkey oak forest, although differences in functional diversity of soil microbial community between management were observed, for the other investigated parameters, the differences were mainly linked to seasonality. The findings highlight that the soil organic matter preservation depends on the type of forest, but the soil microbial community was able to recover after about 15 years from coppice intervention in both forest ecosystems. Thus, the type of management implemented in these forest ecosystems, not negatively affecting soil organic matter pool, preserving microbial community and potentially soil ecological functions, is sustainable in a scenario of climate change.

**Supplementary Information:**

The online version contains supplementary material available at 10.1007/s00248-024-02402-2.

## Introduction

Sustainable Development Goal 15 of Agenda 2030 [1] is about ensuring the protection, restoration and sustainable use of terrestrial ecosystems, reversing land degradation, halting biodiversity loss and sustainably managing forests. From an ecological perspective, forestry interventions can be defined as disturbances actively implemented with the aim of obtaining a variety of forest-based ecosystem services; thus, it is pivotal to know the recovery time post-management disturbance for a sustainable use of forests [2]. Good practices of forest management are important to ensure that forests supply services which sustain life [3] and to increase forest resiliency in a world of increasing pressures [4].

In Europe, the most common forest management systems, classified by the method of tree regeneration [5], are as follows: coppice, stands originating from stool shoots or suckers of vegetative, and high forest, stands predominantly of seedling origin. The two management account for around 42.3 and 41.9% of the Italian forest area, respectively [6]. Thinning of forests is a commonly used management practice [7] to promote natural trees regeneration through increasing the temperatures, microbial activity and irradiance of soil [8], converting coppice forest into high forests. Forest management influences the occurrence of tree species, the maximum stand density and hence the organic matter input to the soil decomposer system through litter [9–13] and, consequently, nutrient stock and C feedbacks [14]. Organic carbon stocks of the forest floor can be reduced if thinning is intense, i.e. up to 50% reduction of basal area compared to unthinned control [3]. Thinning operated on *Quercus ilex* L. caused the almost complete removal of organic horizons, with consequent negative short-term impact on organic carbon content, and the activation of erosion processes [15]. Repeated thinning from below in a Southern European population of Scots pine (*Pinus sylvestris* L.) led to a significant stand biomass and C stock reduction (about 28%) in moderately thinned stands [16]. A study on *Quercus* stands in Spain suggested a positive tree density effect on soil C stocks [17]. However, Camponi et al. [18] highlighted that thinning operated on turkey oak coppice did not affect soil capacity to store C and nutrients.

Given the close relationships among forest canopy, soil characteristics and edaphic community [19], forest management can impact not only the carbon balance in soil, but also the soil microbial community and affect key ecosystem functions it performs [20]. Ananbeh et al. [12] found similar soil specific enzyme activity levels in sessile oak coppice and high forest, suggesting that coppice abandonment has led to the recovery of soil metabolic activity after 90 years, whereas organic matter depletion is a long-term effect that persists. In a Mediterranean oak forest, soil physico-chemical and biological proprieties were negatively affected by coppicing and recovered after 5 years from the disturbance of management [21]. The changes in soil microbial diversity can result in change in the decomposition of different C substrates [22]: rich-species saprotrophic communities potentially lead to a more stable soil organic matter [23].

Moreover, soil microbial properties are also subject to seasonal variations of climatic conditions via direct effects or indirect effects on vegetation [24, 25]. Thus, the effects of the interaction between forest management and climate on soil microbial community are of key importance, although scantly investigated [26–29]. Also, microclimate condition can interact with management, as reported by Grayston et al. [30]: after 1 year, heavy thinning significantly reduced microbial biomass and soil microbial activity in *Fagus sylvatica* L. forest in southern Germany compared to the unthinned control in cool moist condition but not in warm dry condition.

In this study, we compared the potential effect of different forest management, coppice and high forest, on soil microbial functional diversity and other soil properties in two forests (turkey oak and beech) in Southern Italy, during two seasons with different climatic conditions (summer and autumn).

We investigated functional diversity of microbial communities by the community-level physiological profile (CLPP), basing on the carbon consumption patterns, and several enzyme activities linked to biogeochemical cycles of C, N and P, as useful and complementary indicators of the effects of soil changes and disturbances [31, 32]. Since forest coppicing reduces and changes the inputs of organic matter, affecting different soil properties, as well as the size, composition and activity of the soil microbial community [33–35], we hypothesized that coppicing influences soil microbial functional diversity with an overall decrease, especially when microbial community experienced limiting climatic condition. The results will provide useful data for the following: establishing the long-term effects of these forest management practices on soil quality in order to clarify if the reintroduction of coppicing in abandoned coppice forests is a good practice and, improving the sustainability of forest ecosystem management in a scenario of climate change.

## Materials and Methods

### Study Area and Sample Collections

In the National Park of Matese, South Italy, included within the Special Areas of Conservation (SAC) (http://natura2000.eea.europa.eu) “La Gallinola — Monte Miletto — Monti del Matese” (Cod. IT 7222287), two forest sites dominated by European beech (*Fagus sylvatica L*.) and turkey oak (*Quercus cerris* L.), respectively, were selected for the study. These forest categories are selected being the most extended in Matese area.

For soil sampling, particular attention was paid to the uniformity of the lithological substrate which was classified as Pachi-Eutrisilic Andosol [36] for both forest sites. The soil textures were silty sand for soil under beech and as sandy silt for soil under turkey oak (Table 1) according to Shepard classification [37].

**Table 1:** Soils physico-chemical characteristics of beech and turkey oak under different forest management, sampled in two seasons. When present, different lowercase letters indicate significant differences between forest management in each season; different capital letters indicate significant differences between seasons in each forest management (*n* = 9)

Forest system	Texture	WHC % d.w	SOM % d.w	pH	WC % f.w
**Beech**	Silty sand	73 ± 9.3			
Hight forest summer			23 ± 1.1 b B	6.2 ± 0.08	39 ± 1.0 b B
Coppice summer			28 ± 1.7 a	5.8 ± 0.01	44 ± 0.42 a
Hight forest autumn			26 ± 1.9 A	6.6 ± 0.31	45 ± 1.6 A
Coppice autumn			28 ± 2.4	5.6 ± 0.23	45 ± 2.6
**Turkey oak**	Sandy silt	30 ± 8.5			
Hight forest summer			17 ± 2.9	6.9 ± 0.07	16. ± 0.48 a
Coppice summer			16 ± 1.4 A	6.6 ± 0.08	13 ± 0.39 b B
Hight forest autumn			11 ± 0.40 a	7.2 ± 0.45 a	26 ± 1.6
Coppice autumn			9.0 ± 0.50 b B	6.2 ± 0.55 b	23 ± 0.45 A

In each forest site, we identified areas under different management: areas managed by coppicing (C), the last cut was applied at the beginning of the twenty-first century, and undisturbed and unmanaged areas from the beginning of the twentieth century, now converted in high forest (H). Table 2 shows the geographical, topographical and structural characteristics of the high forest and coppice forests.

**Table 2:** Geographical, topographical, and structural characteristics of the high forest and coppice forest dominated by *Fagus. sylvatica* L. (beech) and *Quercus cerris* L. (turkey oak) (*n* = 30)

Forest system	GPS coordinates	Altitude (m.a.s.l.)	Extension (hectares)	Stand age (years)	Tree height (m) Min–max	Stems diameter (cm) Min–max	LAI
**Beech**		800			15–27		
Hight forest	41°25ʹ00.5″N 14°25ʹ53.2″E		99	100		80–200	2.8 ± 0.11
Coppice	41°25ʹ05.2″N 14°25ʹ55.0″E		11	20		20–40	3.3 ± 0.08
**Turkey oak**		200			10—20		
Hight forest	41°23ʹ05.6″N 14°08ʹ24.0″E		126	70–80		20–35	2.9 ± 0.08
Coppice	41°22ʹ56.8″N 14°08ʹ23.2″E		54	15		15–20	2.9 ± 0.08

A completely randomized design consisting of six plots (total surface 150 m^2^) was established in each area (i.e. three plots per management). At each plot, after litter removing, eight soil cores were randomly collected at 0–10 cm depth and then pooled resulting in a homogeneous and representative composite soil sample.

The samplings were carried out in July and October 2022; the summer sampling (S) was characterized by a warmer and drier condition compared to autumn one (A) (http://www.agricoltura.regione.campania.it/meteo/meteo_2022.html).

The experimental design is therefore two-factorial, with the factors “management” and “season”.

Soil samples from each plot were separately analysed, at both summer and autumn samplings. A subset of each composite soil sample was sieved (< 2 mm) and stored at 4 °C for few days until the biological analyses.

### Soil Physico-chemical Analysis

Soil grain size distribution was accomplished by a combination of wet sieving of the > 63-μm fraction and the sedimentation method based on Stokes’ Law, for silt and clay fractions (2 to 63 μm), on oven-dried samples (105 °C for 48 h; MMM Group Ecocell) pretreated with H_2_O_2_, to assure complete removal of organic material, and dispersant solution to remove aggregates [38]. According to the methods of soil chemical analyses [39], soil water holding capacity (WHC) was measured through the gravimetric method, after water saturation and leaching of gravitational water followed by oven drying (105 °C for 48 h); on sieved (< 2 mm) soil samples were determined water content (WC) by the drying method (105 °C until constant weight), soil pH via potentiometric method (SenseION + PH3, Hach) in a water suspension (1:5, w/v soil:water) and soil organic matter (SOM) by calcination in muffle (550 °C for 4 h; Nabertherm GmbH, Controller B 170).

Soil organic carbon (SOC) consists of a variety of compounds with different chemical characteristics and physical availability. The SOC biochemical quality was quantified by a two-step acid hydrolysis approach [40]. Labile fraction could be putatively identified as polysaccharides, derived from plant and microbial sources, and recalcitrant that contains cellulose which is more resistant than labile fraction [40]. Labile soil C was extracted from 250 mg of air-dried soil with 7.5 mL of 2.5 M H_2_SO_4_. The mixture was heated to 100 °C for 30 min in a digestion block. To extract the recalcitrant part of the soil C, the soil from the previous extraction, containing the unhydrolysed residues, was dried at 60 °C and, after cooling, 0.6 mL of 13 M H_2_SO_4_ was added; then, the tubes were shaken overnight at room temperature. Thereafter, deionized water was added to dilute the acid to 1 M, and the residues were hydrolysed for 3 h at 100 °C. After centrifugation at 3000 × g for 4 min, the clear hydrolysate was collected. Labile and recalcitrant C concentrations were measured by spectrophotometric analysis at 254 nm (UV–VIS Jenway 6715 Spectrophotometer), adapting the Standard Methods 5910 B [41]. The stable C residue was analysed with a CHNS elemental analyser (Carlo Erba 1500).

### Soil Biological Parameters

Six enzymatic activities were analysed by spectrophotometric method: arylsulfatase activity [42] linked to S cycle, β-glucosidase activity [43] and laccase activity [44] linked to C cycle, phosphatase activity [42] linked to P cycle, β-glucosaminidase activity [45] linked to N cycle and hydrolase activity [46] as potential total enzymatic activity. Briefly, 0.1 g sieved (< 2 mm) soil was incubated at 37 °C (25 °C for hydrolase activity) for 30 min under shaking with respective substrates at the optimal pH for the specific enzymatic reaction. Hydrolase activity was calculated from a fluorescein calibration curve at 490 nm. The arylsulfatase, β-glucosidase, phosphatase and β-glucosaminidase activities were calculated from a p-nitrophenol calibration curve at 410 nm. For laccase activity, the absorbance of the reaction product (ABTS +) was measured at 420 nm.

Functional diversity of microbial communities based on the carbon consumption patterns (community-level physiological profile CLPP) was investigated applying the Biolog® eco-plates method. The measurements were carried out as described by [47] with some optimizations as described by [48]. Four grams of soil was suspended in 36 mL 0.85% sterile NaCl solution and shaken for 30 min. Serial dilution was made with 0.85% sterile NaCl solution; 150 μL of the 10^−3^ dilution was added to the eco-plate wells and then incubated at 25 °C. The absorbance was measured with Bio-Rad Microplate Reader (Model 680) at every 24 h for 192 h at 600 nm wavelength.

Triplicate analyses were conducted for each sample.

### Data Analysis

The Biolog® eco-plates data measured at 192 h (8 days) were used to calculate Simpson diversity (*D*), Shannon diversity (*H*ʹ), functional diversity and the average well colour development (AWCD):

- Simpson diversity D = 1 − Σ*P*_*i*_.^2^
- Shannon diversity *H*ʹ = − Σ*P*_*i*_ln*P*_*i*_
  with *P*_*i*_ = *n*_*i*_/*N.*
  where the *P*_*i*_ is the ratio colour development of the *i*th well compared to the total colour development of all the wells, *n*_*i*_ is the absorbance value for a specific substrate and *N* is the sum of all the positive absorbance values for the plate. Simpson index ranges from 0 to 1; the larger the value of *D*, the higher the physiological diversity of bacterial communities. Shannon index ranges from 0 to ln of the number of substrates; the higher the value of *H*, the more metabolically active microbial communities are able to degrade more substrates.
- Functional Diversity (%) = *p*/*N* × 100
  where *p* is number of positive (purple/pink) carbon source wells and *N* is the number of substrates; in this case, *N* = 31. This value varies from 0 to 100%, being 0 low and 100% high diversity.
- Average well colour development AWCD = Σ *A*_*i*_/*N* [49]
  where *A*_*i*_ is the absorbance value of each carbon source containing well corrected with blank and *N* is the number of substrates; in this case, *N* = 31.

To analyse AWCD of all carbon sources, the substrates were grouped into six categories representing different substrate groups according to Sala et al. [50]: amino acids, amines, carbohydrates, carboxylic acids, polymers and miscellaneous. Heat map of the metabolic profile of microorganisms under beech and turkey oak based on the consumption of various carbon sources after 192 h of incubation using the EcoPlate method and the cluster analysis of AWCD were performed.

To test the response of the biological and chemical parameters against the forest management and season (fixed factors), the permutation analysis of variance (PERMANOVA) was applied. *P*-values were calculated using the Monte Carlo permutation test (999 permutations) together with the permutation of residuals under an unrestricted permutation model. We used omega-squared (*ω*^2^) to incorporate the effect size in PERMANOVA tests made for each site.

If the PERMANOVA showed significant differences for forest management and season interaction, we proceeded with two-way repeated measurements ANOVAs to deepen the differences in the biological and chemical parameters, using forest management and season as fixed factors. Data were ln- and square root-transformed when needed to fulfil the assumptions of the ANOVA. Post hoc Tukey’s HSD test (*P* = 0.05) was used for comparisons between forest management in each season and between seasons for each forest management.

Soil physico-chemical, biological characteristics and functional diversity, as well as the AWCD, Simpson and Shannon indices in the two forest systems were processed by means of two separated Q-type principal component analyses (PCA). The “vegan”, “tidyverse”, “effectsize”, “dplyr “, “tibble”, “purr”, “nlme”, “multcomp”, “emmeans”, “factoextra”, “ggplot2”, “ggpubr” and “GGally” packages in the R 4.1.2 programming environment [51] were used.

## Results

Table 1 shows physico-chemical characterises of investigated soils. In beech soil, SOM content and WC were higher (*P* < 0.01) under coppice management respect hight forest in summer; conversely, in turkey oak, SOM and WC were higher (*P* < 0.01) in hight forest management respect coppice in autumn and summer, respectively. Seasonal differences were observed for beech under high forest and for turkey oak under coppice management.

The PERMANOVA (*ω*^2^ = 0.76) highlighted significant differences between forest management (*F* = 7.3, *p* < 0.001) and seasons (*F* = 11, *p* < 0.0001), as well as for their interactions (*F* = 4.1, *p* < 0.01) for soil under beech; for soil under turkey oak, the PERMANOVA (*ω*^2^ = 0.50) highlighted significant differences between management (*F* = 2.9, *p* < 0.05) and seasons (*F* = 4.6, *p* < 0.01).

**Fig. 1:**
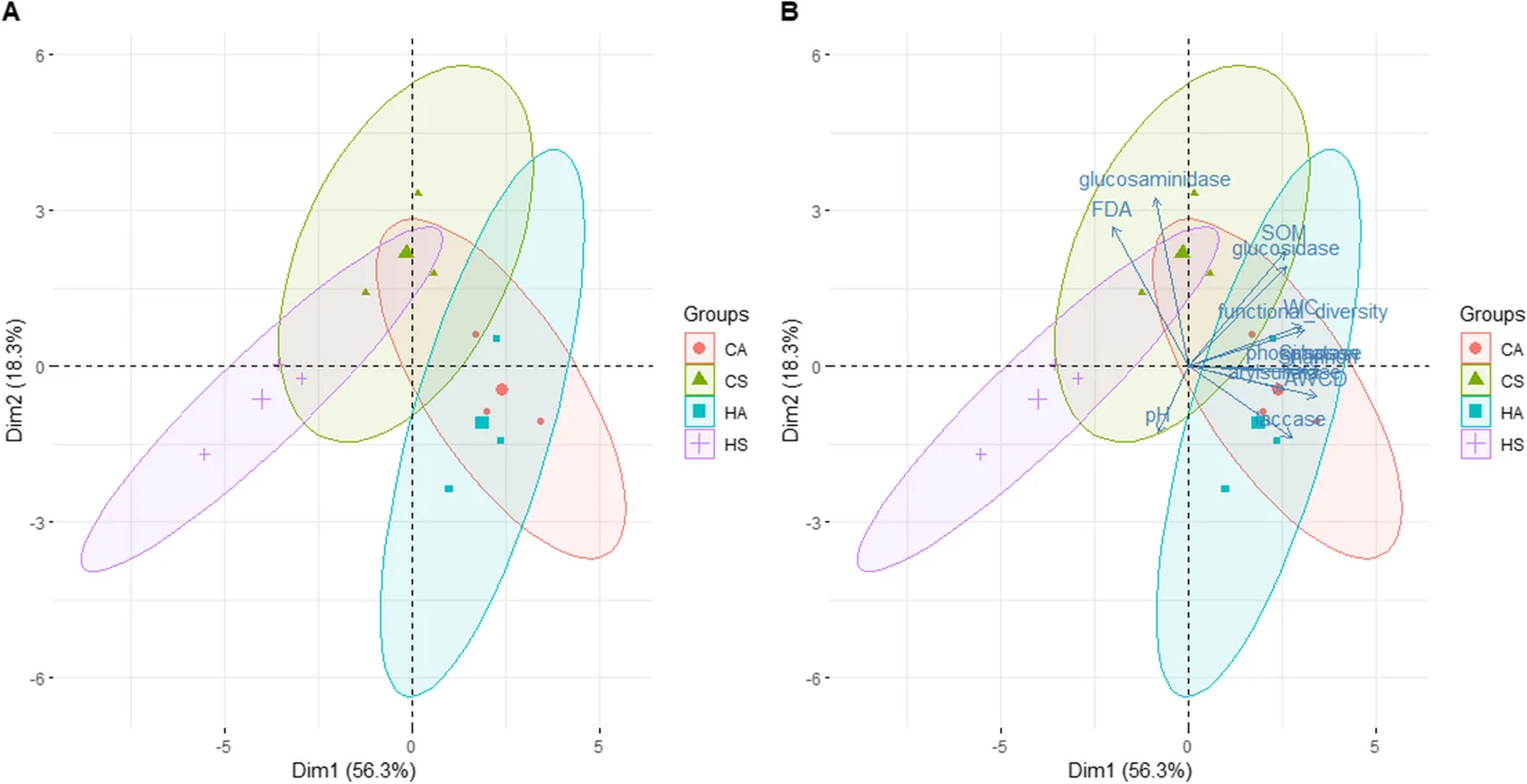
Biplots of principal component analyses (PCA) with **A** the superimposition of the confidence ellipses showing the differentiation between the interaction forest management and season factors in soil under beech. The plots display the first (PC1) and second (PC2) principal components, variance explained by PC1 and PC2, and **B** the variable contributions to the first two principal components. HA = high forest autumn; CA, coppice autumn; HS, high forest summer; CS, coppice summer

**Fig. 2:**
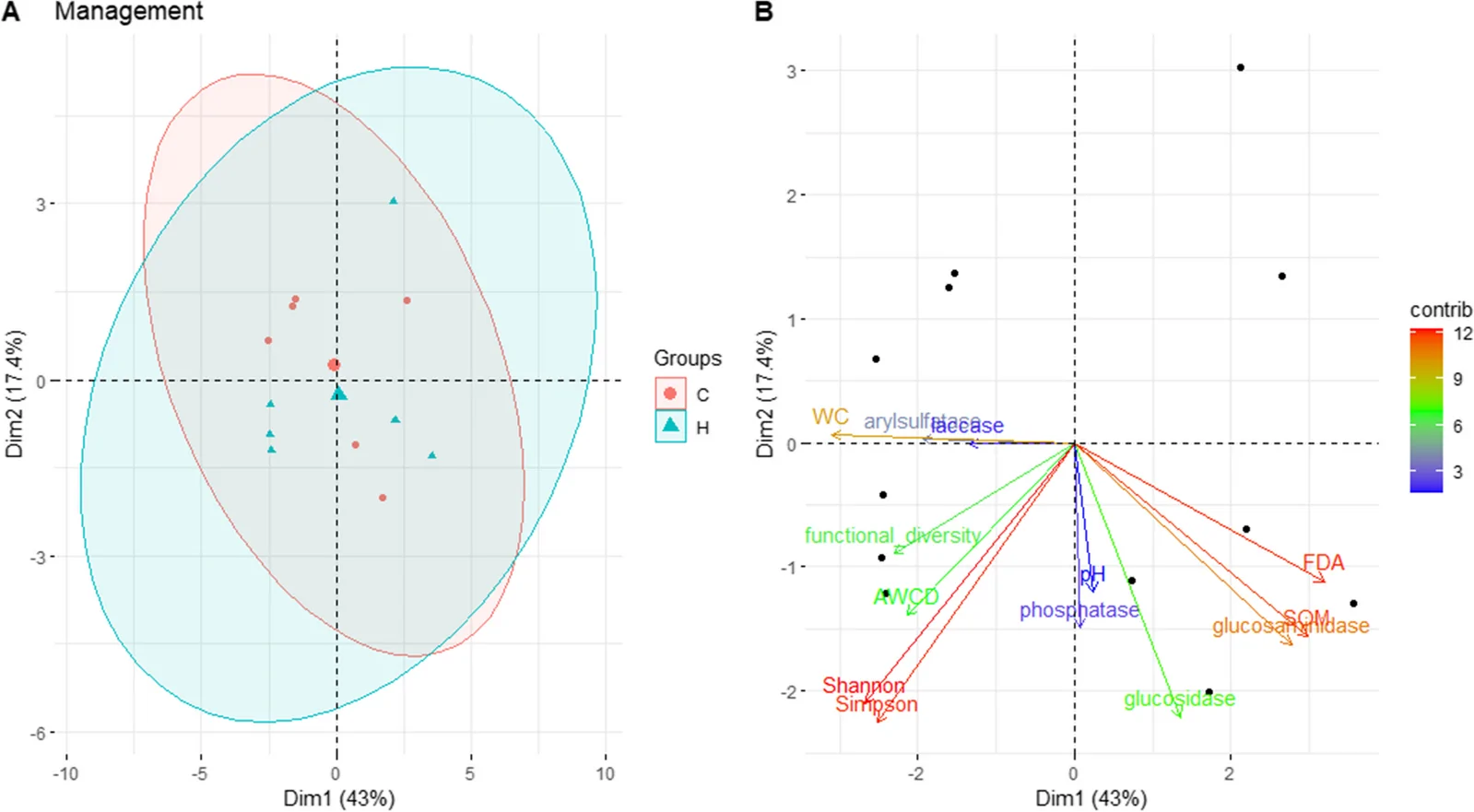
Biplots of principal component analyses (PCA) with **A** the superimposition of the confidence ellipses showing the differentiation between forest management in soil under turkey oak. The plots display the first (PC1) and second (PC2) principal components, the variance explained by PC1 and PC2, and **B** the variable contributions to the first two principal components. H, high forest; C, coppice

**Fig. 3:**
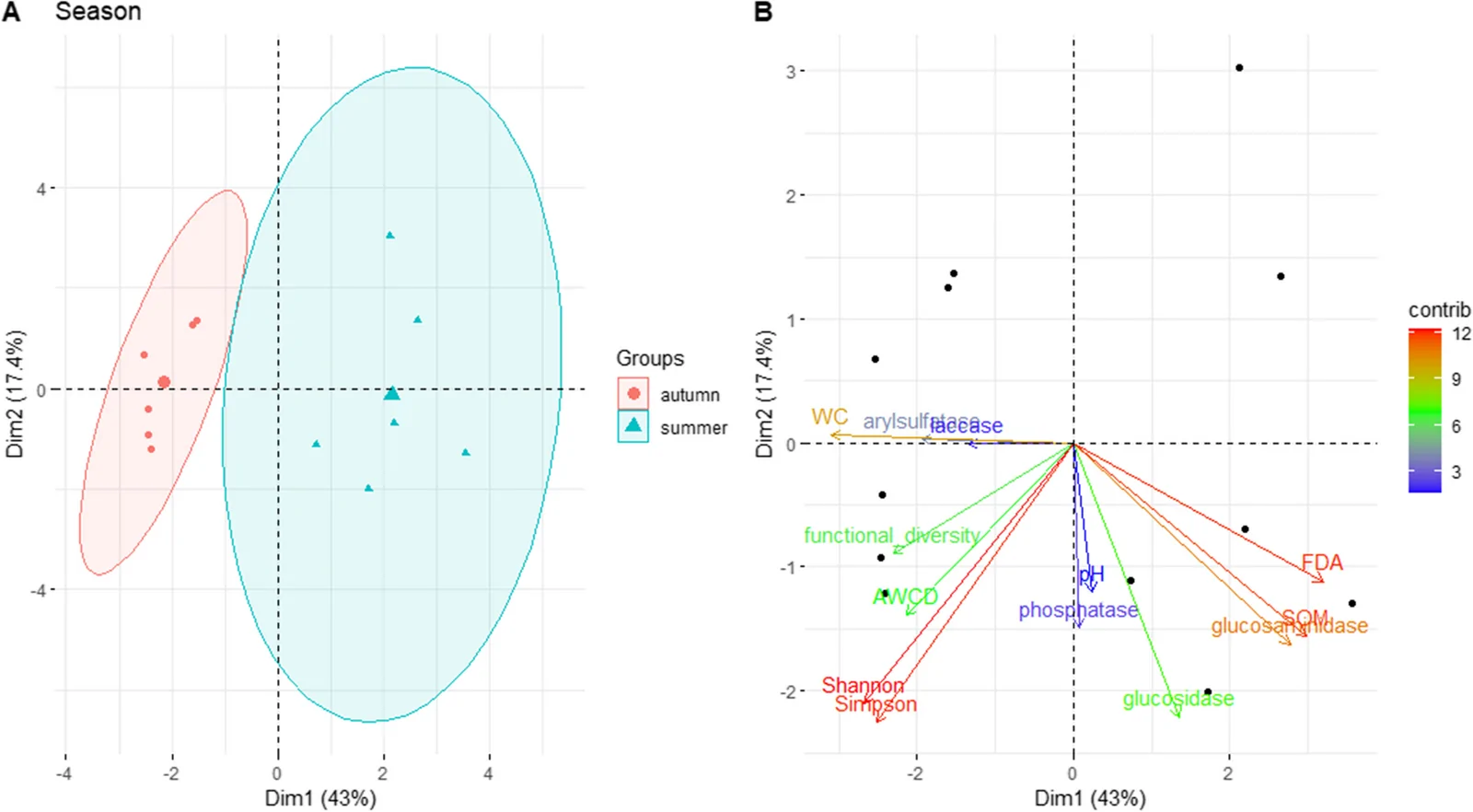
Biplots of principal component analyses (PCA) with **A** the superimposition of the confidence ellipses showing the differentiation between seasons in soil under turkey oak. The plots display the first (PC1) and second (PC2) principal components, variance explained by PC1 and PC2, and **B** the variable contributions to the first two principal components

PCA shows the separation between the forest management and seasons in relation to physico-chemical and biological characteristics. For soil under beech (Fig. 1), the two principal components account for 56.3 and 18.3% of the variation, respectively. PC1 axis separates the two seasons (HS and CS in respect to HA and CA), and PC2 axis separates CS from the others. The two principal components of the PCA soil under turkey oak (Figs. 2 and 3) account for 43 and 17.4% of the variation, respectively, and we found a clear separation for the two seasons (Fig. 3).

**Fig. 4:**
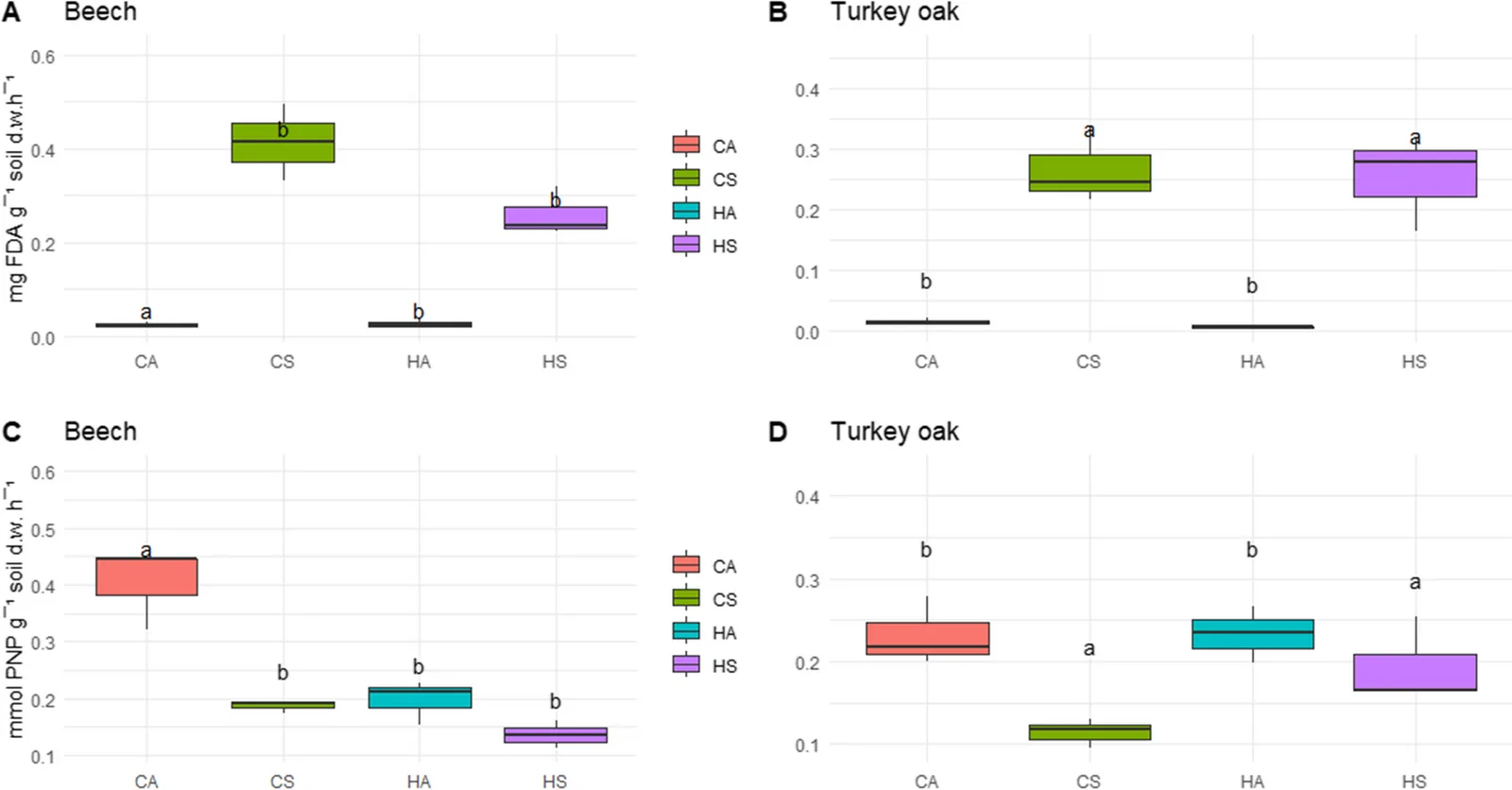
FDA activity (mean ± s.e.) (upper panel) in soil of **A** beech and **B** turkey oak and arylsulfatase activity (mean ± s.e.) (lower panel) in soil of **C** beech and **D** turkey oak under different forest management, sampled in two seasons. H, high forest; C, coppice; A, autumn; S, summer. Different letters indicate significant differences between forest management

The most important variables in differentiating between the soil forest management and season were defined as those which had at least half of their variance explained by PC 1 or PC 2 and were different depending on the forest system. In beech forest soil, the most important variables in differentiating between seasons (Fig. S1a) were as follows: Simpson, Shannon, AWCD indices and % functional diversity. Indeed, these indices were significantly lower in summer (Table 3). The most important variables in differentiating between management (Fig. S1b) were as follows: glucosaminidase and FDA activities, SOM and glucosidase activity. Indeed, in summer, FDA activity (Fig. 4a) and SOM (Table 1) were significantly higher under coppice management compared to hight forest.

**Table 3:** Diversity indexes and total AWCD (at 192 h) of soil microbial community in beech and turkey oak under different forest management, in two seasons. When present, different lowercase letters indicate significant differences between forest management in each season; different capital letters indicate significant differences between seasons in each forest management (*n* = 9)

Forest system	Shannon	Simpson	Functional diversity (%)	AWCD (total C utilization)
**Beech**				
Hight forest summer	1.40 ± 0.020 b B	0.958 ± 0.002 b B	91.4 ± 3.72 B	1.10 ± 0.050 b B
Coppice summer	1.44 ± 0.006 a	0.962 ± 0.001 a	95.7 ± 1.86	1.33 ± 0.080 a
Hight forest autumn	1.46 ± 0.002 A	0.9645 ± 0.0003 A	96.8 ± 0.001 A	1.50 ± 0.042 A
Coppice autumn	1.45 ± 0.020	0.963 ± 0.002	95.7 ± 1.86	1.49 ± 0.207
**Turkey oak**				
Hight forest summer	1.45 ± 0.007 B	0.964 ± 0.001 B	96.8 ± 3.22	1.33 ± 0.020 b B
Coppice summer	1.46 ± 0.009	0.965 ± 0.001	96.8 ± 3.23	1.54 ± 0.130 a
Hight forest autumn	1.47 ± 0.002 A	0.966 ± 0.001 A	100 ± 0.00	1.57 ± 0.021 A
Coppice autumn	1.47 ± 0.003	0.965 ± 0.001	98.9 ± 1.87	1.50 ± 0.064

In turkey oak forest soil, the most important variables in differentiating between seasons (Fig. S2a) were as follows: FDA, WC, SOM, glucosaminidase activity, Simpson and Shannon indices. Indeed, FDA activity (Fig. 4b) and SOM (Table 1) under coppice management were significantly higher in summer compared to autumn, while Simpson and Shannon indices under hight forest management were significantly higher in autumn respect summer.

Among the other enzymatic activities measured, the arylsulfatase activity (Fig. 4c, d) in soil under turkey oak showed significant differences between the different management in summer, while the activity for both forests was higher in coppice management compared to high forest in autumn.

Regarding the organic carbon fractions, soil under beech showed significant differences (*p* < 0.01) between the different management in summer for recalcitrant and stable C, with values higher in soil under coppice management compared to high forest (Table 4). Soil under turkey oak showed mainly significant difference (*p* < 0.001) between the two seasons: both labile and recalcitrant C were higher in autumn respect summer, while stable C was higher in summer respect autumn (Table 4).

**Table 4:** Labile C, recalcitrant C, and stable C in soils of beech and turkey oak under different forest management, sampled in two seasons. Concentrations are expressed as milligrams per kilogram of soil (mean ± s.e.). When present, different lowercase letters indicate significant differences between forest management in each season; different capital letters indicate significant differences between seasons in each forest management (*n* = 9)

Forest system	Labile C	Recalcitrant C	Stable C
**Beech**			
Hight forest summer	27 ± 0.77	4.3 ± 0.45 bB	60 ± 22 b
Coppice summer	23 ± 0.69 B	5.4 ± 0.92 aB	101 ± 12 a
Hight forest autumn	26 ± 4.5	6.1 ± 0.15 A	91 ± 32
Coppice autumn	28 ± 1.5 A	6.9 ± 0.32 A	94 ± 6.6
**Turkey oak**			
Hight forest summer	16 ± 1.7 B	3.3 ± 0.54 B	83 ± 22 aA
Coppice summer	17 ± 0.98 B	2.9 ± 0.06 B	41 ± 2.1 bA
Hight forest autumn	21 ± 1.0 A	4.2 ± 0.46 A	33 ± 17 B
Coppice autumn	22 ± 1.6 A	3.9 ± 0.24 A	20 ± 1.0 B

On average, AWCD (Table 3) was higher in autumn in both forest soils, but only in summer, the management type showed a significant difference, stimulating the microbial community of soil under coppice management. In beech hight forest, the soil microbial functional diversity was higher in autumn compared to summer. Shannon and Simpson indexes showed significantly different values between the forest management in soil under beech, with higher value in coppice compared to high forest management.

**Fig. 5:**
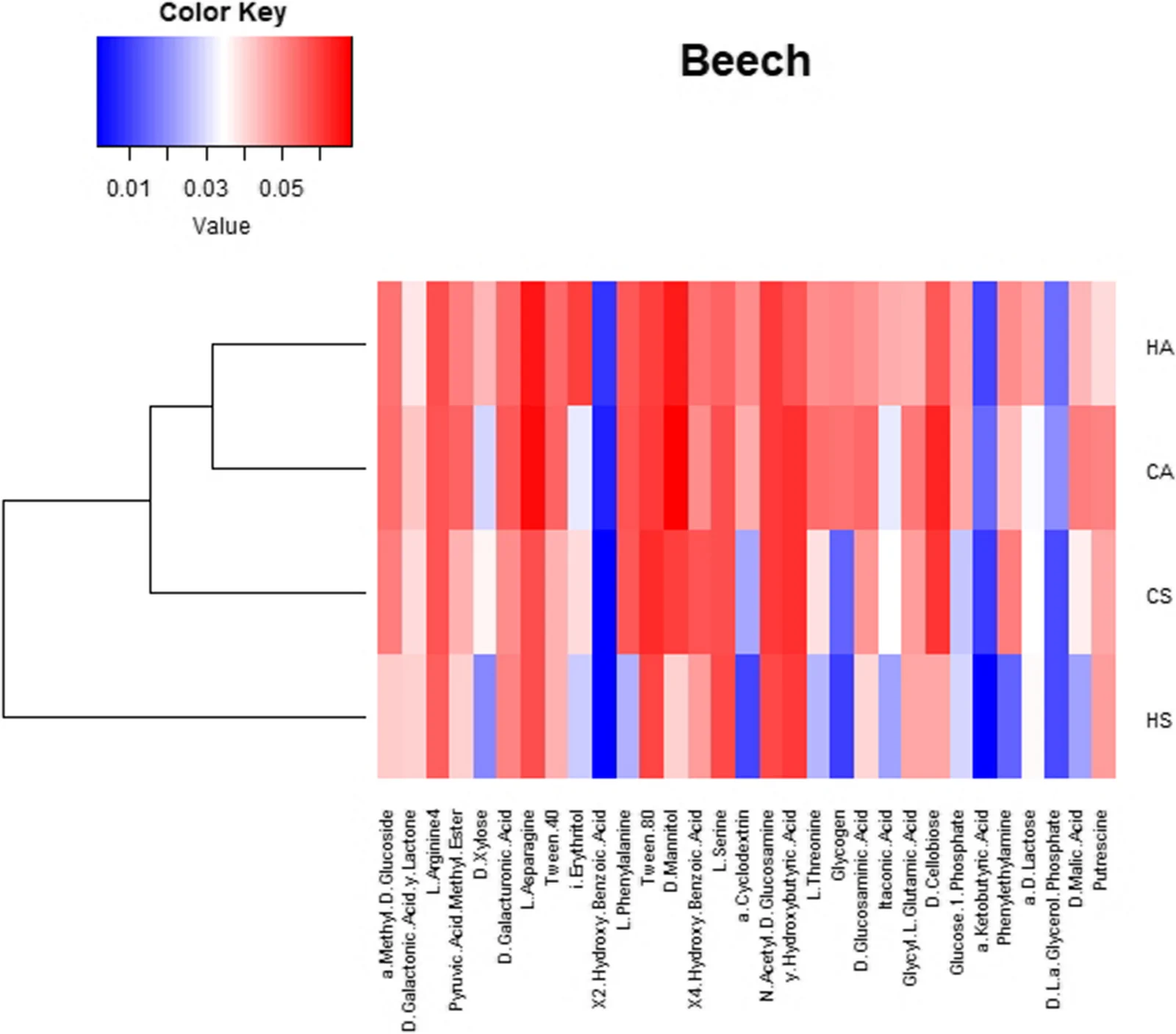
Heat map of the metabolic profile of microorganisms based on the consumption of various C sources after 192 h of incubation (EcoPlate method) in soils of beech forest under different forest management, sampled in two seasons. In the heat map analysis, the AWCD scores (absorbance of the carbon substrate − absorbance of the control well)/number of carbon substrates are shown. The highest consumption (i.e. higher AWCD) can be identified by a red colour and the lowest consumption (i.e. lower AWCD) by a blue colour. H, high forest; C, coppice; A, autumn; S, summer

**Fig. 6:**
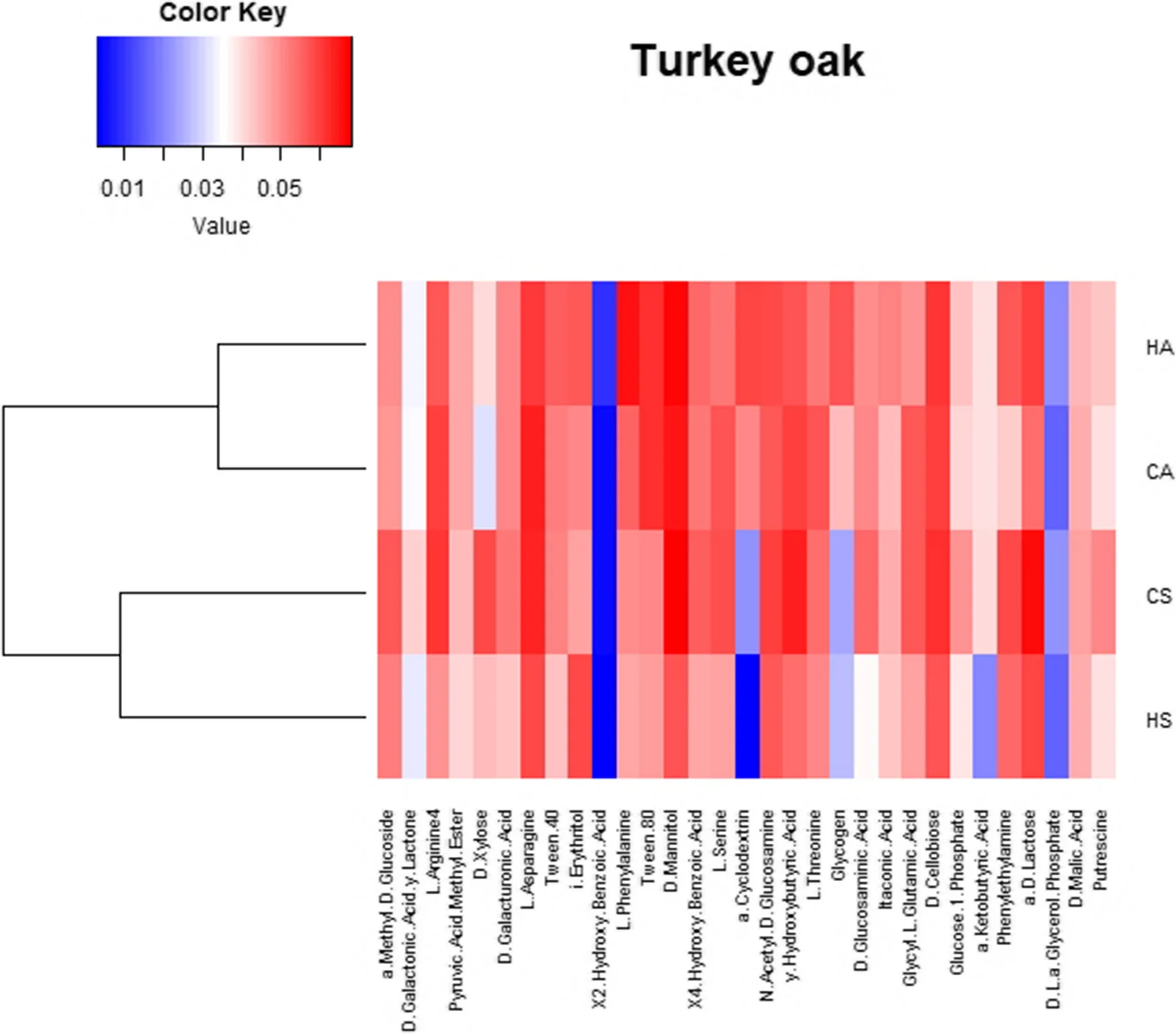
Heat map of the metabolic profile of microorganisms based on the consumption of various C sources after 192 h of incubation (EcoPlate method) in soils of turkey oak forest under different forest management, sampled in two seasons. In the heat map analysis, the AWCD scores (absorbance of the carbon substrate − absorbance of the control well)/number of carbon substrates are shown. The highest consumption (i.e. higher AWCD) can be identified by a red colour and the lowest consumption (i.e. lower AWCD) by a blue colour. H, high forest; C, coppice; A, autumn; S, summer

CLPP analysis allowed to recognise different carbon substrate utilization by soil microbial community in forests under different management practices. The degree of each carbon substrates consumption is visualized in the form of a heat map (Figs. 5 and 6). Visual analysis of the heat map showed that in autumn, the microorganisms in both beech and turkey oak forest soils had the most intensive utilization of C across all C sources under high forest compared to coppice management. In summer, microorganisms were more stimulated in C utilization in soil under coppice compared to high forest management.

The percentage contribution of C source groups in total C utilization by soil microorganisms (Table 5) was different depending on the management: in summer, microorganisms in soil under beech coppice utilized more intensely amines and amides compared to high forest management. While in soil under turkey oak coppice, microorganisms had the most intensive utilization of amino acids compared to high forest in autumn. In addition, a difference between seasons was evident: under high forest management microorganisms had most intensive utilization of carbohydrates in summer respect autumn, conversely for polymers.

**Table 5:** Percentage contribution of C source groups in total C utilization by microorganisms in soils of beech and turkey oak under different forest management, sampled in two seasons. When present, different lowercase letters indicate significant differences between forest management in each season; different capital letters indicate significant differences between seasons in each forest management (*n* = 9)

Forest system	Amines and amides	Amino acids	Carbohydrates	Carboxylic acids	Polymers	Miscellaneous
**Beech**						
Hight forest summer	6.0 ± 0.30 b	24 ± 1.9	25 ± 1.7	26 ± 1.1	11 ± 1.6	7.0 ± 0.30
Coppice summer	7.0 ± 0.50 a	24 ± 0.80	26 ± 2.4	26 ± 1.6	11 ± 2.4	6.0 ± 1.3
Hight forest autumn	6.0 ± 0.60	22 ± 0.66	26 ± 0.59	24 ± 1.6	14 ± 1.3	6.0 ± 0.60
Coppice autumn	6.0 ± 0.5	23 ± 1.8	23 ± 1.4	25 ± 1.8	14 ± 1.6	6.0 ± 0.60
**Turkey oak**						
Hight forest summer	7.0 ± 0.80	23 ± 1.2	29 ± 1.8 A	25 ± 1.7	9.0 ± 1.0 B	7.0 ± 0.79
Coppice summer	7.0 ± 0.30	22 ± 0.1	27 ± 0.8	26 ± 1.7	9.0 ± 2.0	7.0 ± 0.10
Hight forest autumn	6.0 ± 0.20	21 ± 0.28 b	25 ± 0.40 B	25 ± 0.21	15 ± 0.70 A	6.0 ± 0.19
Coppice autumn	5.0 ± 0.89	23 ± 0.85 a	24 ± 1.4	26 ± 2.2	14 ± 1.7	5.0 ± 0.60

**Fig. 7:**
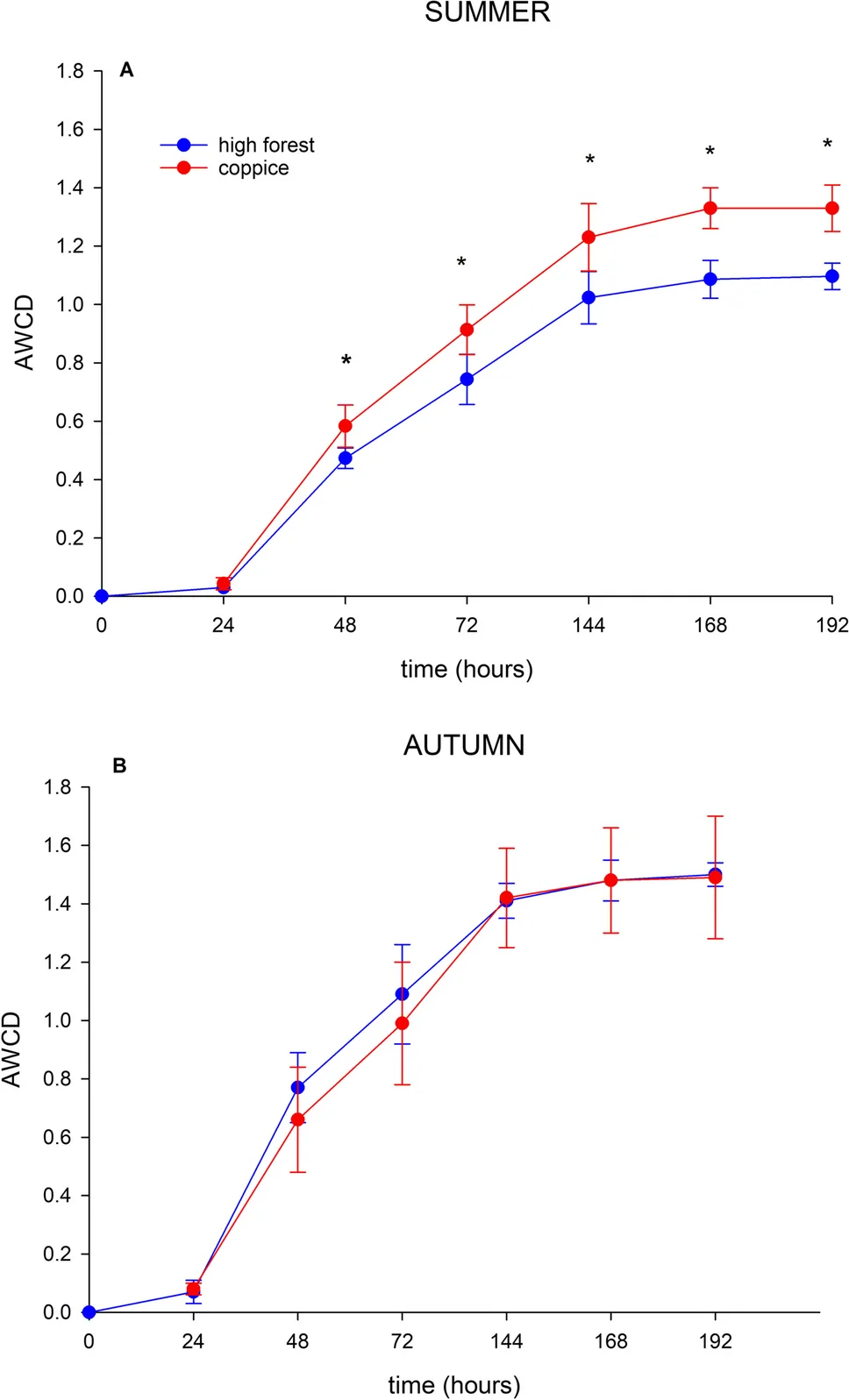
Biolog-AWCD values of microbial communities calculated during a 192-h incubation in soils of beech forest under different forest management, sampled in two seasons: **A** summer and **B** autumn. Asterisks indicate significant differences between forest management

**Fig. 8:**
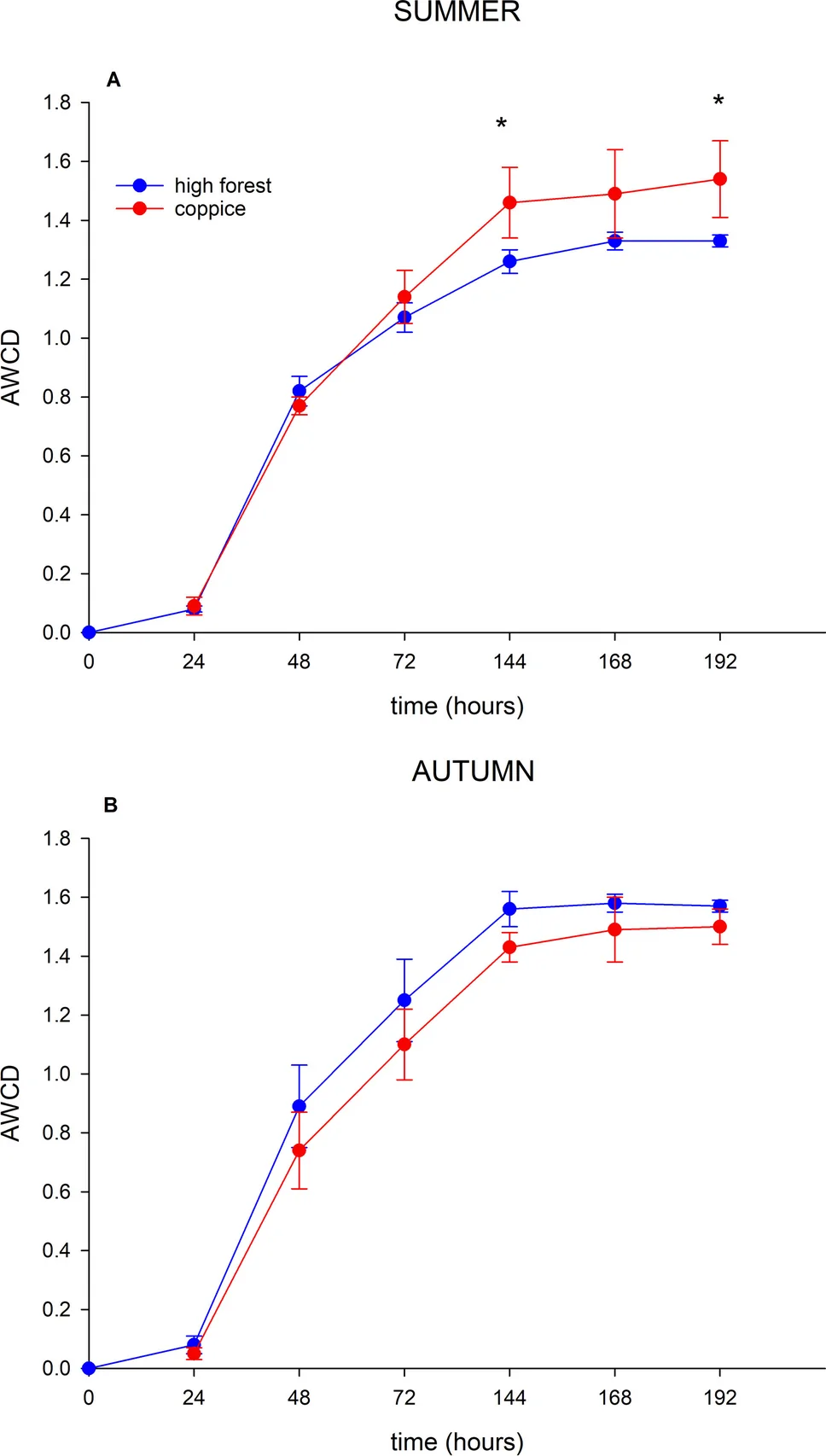
Biolog-AWCD values of microbial communities calculated during a 192-h incubation in soil of turkey oak forest under different forest management, sampled in two seasons: **A** summer and **B** autumn. Asterisks indicate significant differences between forest management

Figures 7 and 8 show Biolog-AWCD values for soil microbial communities under both beech and turkey oak, calculated during 192 h incubation. Microbial activity, as measured by AWCD, in soil under beech continued to increase with time and was significantly higher for coppice compared to high forest management in summer (Fig. 7a) along the time. In autumn (Fig. 7b), soil microbial activity curve did not show significant differences between forest management. Microbial activity also in soil under turkey oak increased with time and was higher for coppice compared to high forest management in summer only after 192 h incubation (Fig. 8a); on the other hand, in autumn (Fig. 8b), the microbial activity was higher in soil under high forest management, although without significant differences compared to coppice.

## Discussion

AWCD is an indicator of the general potential metabolic activity of the microbial community; thus, it is an index of the total bioactivity [52, 53]. AWCD values can be subdivided into groups based on substrate guilds (carbon sources) of similar chemical nature (e.g. amino acids, amines, carbohydrates, carboxylic acids, phenolic compounds and polymers) to assess the potential of the soil microbial community to degrade these carbon sources [50, 53]. The Shannon diversity index (*H*) is used to calculate the physiological diversity of microbial communities [54]. Microbial communities that are able to degrade more substrates or/and to degrade them with similar efficiency would have higher values of *H* compared to the community which is not metabolically active and is not capable of growing in plate conditions [55].

Contrary to our hypothesis, in summer, the AWCD was higher in both coppice forests. In fact, generally the AWCD index is higher in high forests [33–35]. Thus, we can think of a resilience response of the microbial communities in the soil after cutting, which occurred 15–20 years ago, depending on the forest system. Also, Ananbeh et al. [12] found that the recovery of soil metabolic activity occurred 90 years after cutting. Brais et al. [56] and Pignataro et al. [57]observed that the greater part of changes in nutrient cycling occurred during the first years after management. Immediately after coppicing, the increased inputs of litter, plant remains and root exudates to the soil are expected to increase soil organic matter accumulation and modify decomposition processes in the medium to long term. Over time, coppice reduces and changes the inputs of organic matter as well as composition and activity of the soil microbial community. After a time that depends on the type of ecosystem, the soil microbial communities and their activities recover from the negative effects of coppicing and tend to approach the levels found in high forests [58, 59].

In our study, an indirect effect of seasonality on microbial metabolism can be suggested through changes in root exudates and leaf litter due to the new herbaceous and shrubby plant cover colonizing the coppiced plots (observational data). Indeed, in summer, the richer undergrowth compared to autumn (data not shown) favours the microbial metabolism, despite dryer soil conditions than in autumn; furthermore, the content of SOM differed depending on forest management and systems (Table 1).

Organic matter was higher in summer in the soil under coppice beech, while in autumn, it was higher under turkey oak in high forest regime compared to coppice. We can explain the separation between forest beech management with the different contents of SOM and the different concentrations of recalcitrant and stable organic carbon (Table 4). SOM, recalcitrant and stable organic carbon were higher in coppice compared to high forest in summer, and this can explain the higher AWCD and Shannon indices in coppice beech, which indicate high diversity and high metabolic activity. A model focused on the relation among soil microbial functional diversity, chemistry of decomposition products and stability of organic matter was proposed: a high diversity generates a more stable organic matter [23]. The strong correlation found between the functional diversity indices, AWCD and % functional diversity with SOM and organic C in beech system (Fig. S3a) highlights the strong link between the quantity of organic substance and the soil microbial community. This can also explain the most intensive utilization of C sources that in summer were higher in soil under coppice regime compared to high forest. The difference between the two forest management in summer is probably more closely related to the organic matter quality and the greater increase in microbial activity when the temperature was favourable. Indeed, in the summer of 2022, the recorded temperatures (mean temperature in July was 27 °C) were much more favourable for microbial activity than in the autumn (mean temperature in October was 17 °C) (http://www.agricoltura.regione.campania.it/meteo/meteo_2022.html).

In soil under turkey oak, although differences in functional diversity of soil microbial community between management were observed, for the other investigated parameters, the differences were mainly linked to seasonality. Thus, the most intensive utilization of C across all C sources in coppice compared to high forest management was not due to the content of organic carbon, but likely to other kinds of organic compound (e.g. rhizodepositions, including exudates from plant roots and associated symbionts mycorrhizal fungal mycelia), as well as faecal material and bodies of the soil biota [3] and/or the age of the stands. Also the trend of correlation was not evident in the soil under turkey oak (Fig. S3b), in which in fact the separation observed was mainly linked to seasonal differences (Fig. 3a).

Regarding the measured enzymatic activities, we did not find significant differences between the management, except for FDA and arylsulfatase, likely thanks to a resilience response of microbial communities after 15 years. For example Pignataro et al. [57] found an opposite trend, with an increase of almost all enzyme activities probably because the study was performed only after 3 years from coppice.

## Conclusions

In this study, we found, in beech forest under coppice management, a higher content of soil organic matter (but also of soil recalcitrant and stable organic carbon) compared to high forest and this can explain the higher soil microbial functional diversity and metabolic activity. In turkey oak forest, although differences in functional diversity of soil microbial community between management were observed, for the other investigated parameters, the differences were mainly linked to seasonality.

The findings highlight the SOM preservation depends on the type of forest, but the soil microbial community was able to recover after 15 years from coppice intervention in both forest ecosystems. Thus, the type of management implemented in these forest ecosystems does not negatively affect SOM pool, preserves microbial community and potentially soil ecological functions and can be considered as sustainable in a scenario of climate change.

With our results, we can suggest that the reintroduction of coppicing in abandoned coppice forests with these frequencies of cutting is a sustainable intervention in both these forest ecosystems. Research in this direction is needed to identify more clearly forest ecosystems at risk of soil C depletion and loss of microbial functions as well as management practices that do not exacerbate this risk.

## Supplementary Information

Below is the link to the electronic supplementary material.

Supplementary file1 (DOCX 117 KB)

## Data Availability

No datasets were generated or analysed during the current study.
