# Supplementary material for: Recover of Soil Microbial Community Functions in Beech and Turkey Oak Forests After Coppicing Interventions
Source: Microb Ecol. 2024 Jun 28;87(1):86. doi: 10.1007/s00248-024-02402-2 (PMC11213729; doi:10.1007/s00248-024-02402-2)
Supplement: Supplementary file 1 — Supplementary file1 (DOCX 117 KB) [file 248_2024_2402_MOESM1_ESM.docx]

Supplementary figures

**Fig S1.** Variance explained by a) Dim1 and b) Dim2 of the soil physico-chemical and biological variables processed by means of two separated Q-type principal component analyses (PCA) in soil under beech.

a)


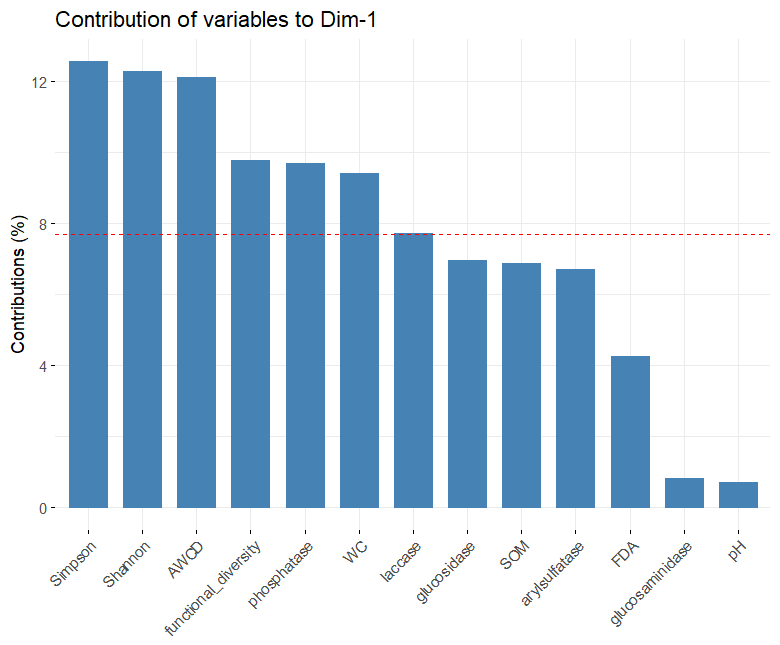


b)


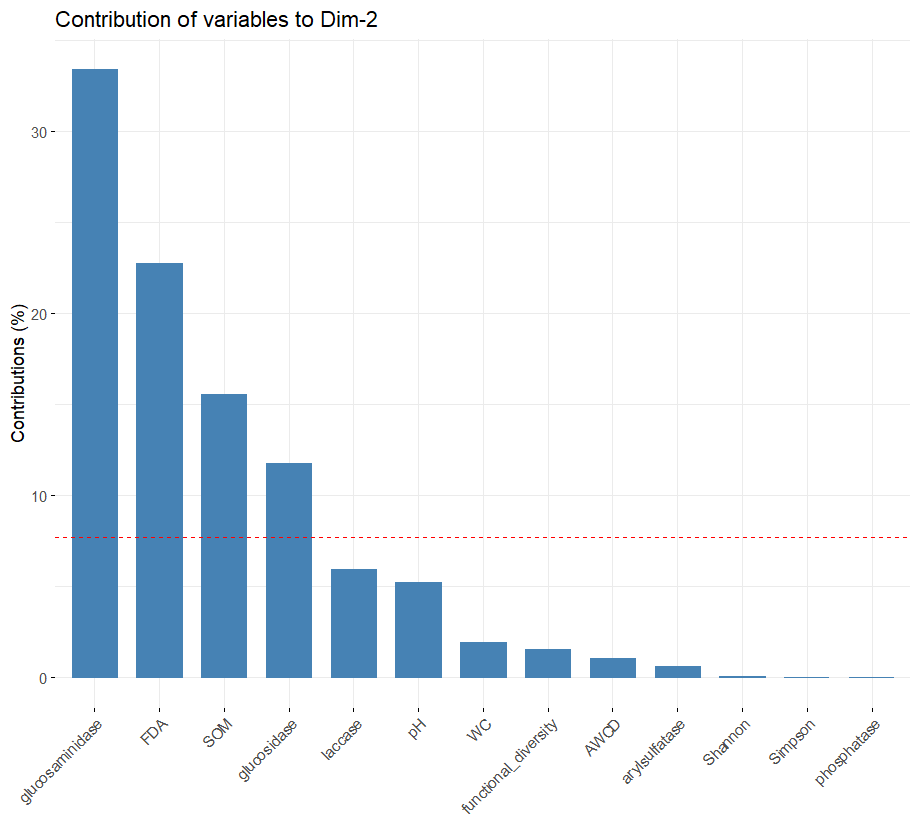


**Fig S2.** Variance explained by a) Dim1 and b) Dim2 of the soil physico-chemical and biological variables processed by means of two separated Q-type principal component analyses (PCA) in soil under turkey oak.

a)


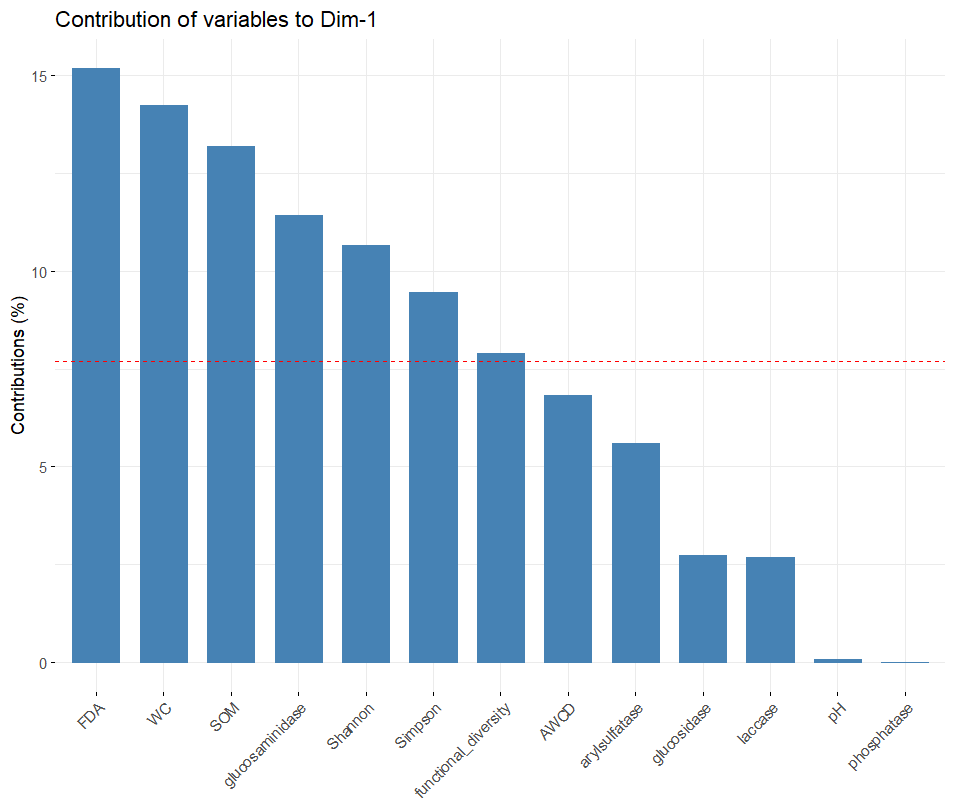


b)


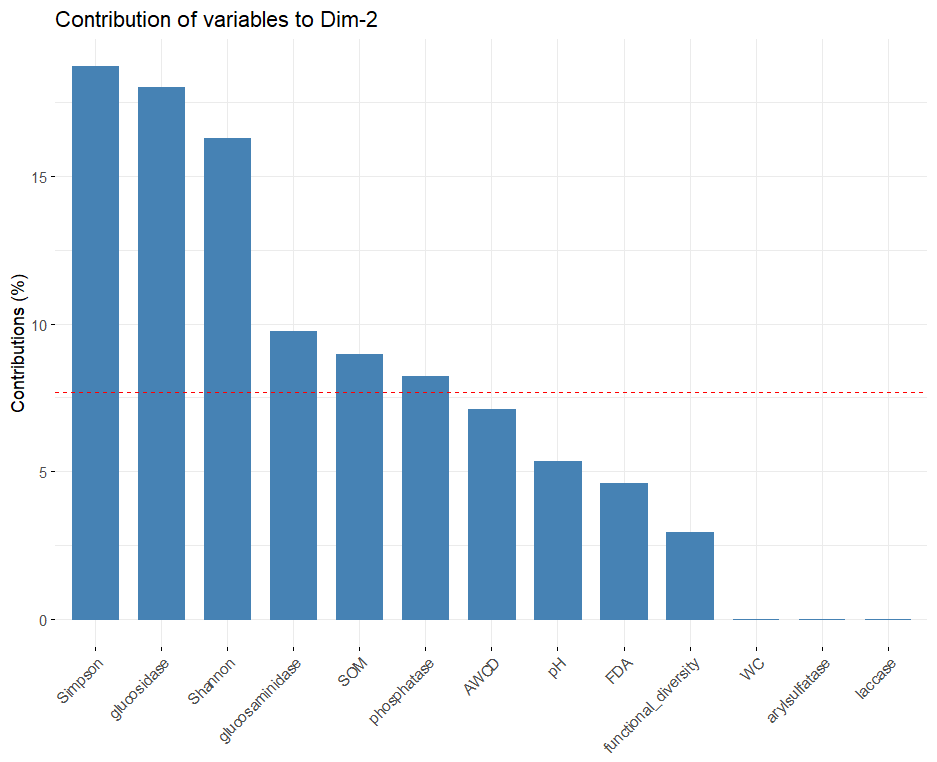


**Fig S3.** Correlation matrices among diversity indices, enzymatic activities and soil properties measured in soil under a) beech and b) turkey oak. A two-tailed correlation analysis was performed (Pearson critical value was 0.250) using the ´corrplot´ function as a graphical output in R environment (R Core Team, 2021).

a)


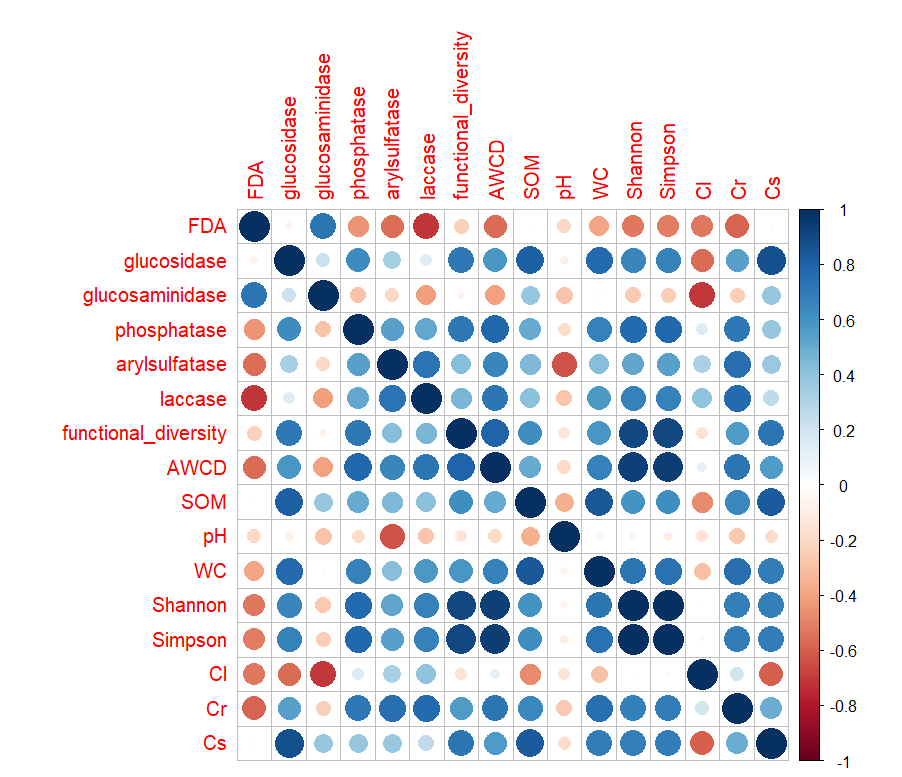


b)


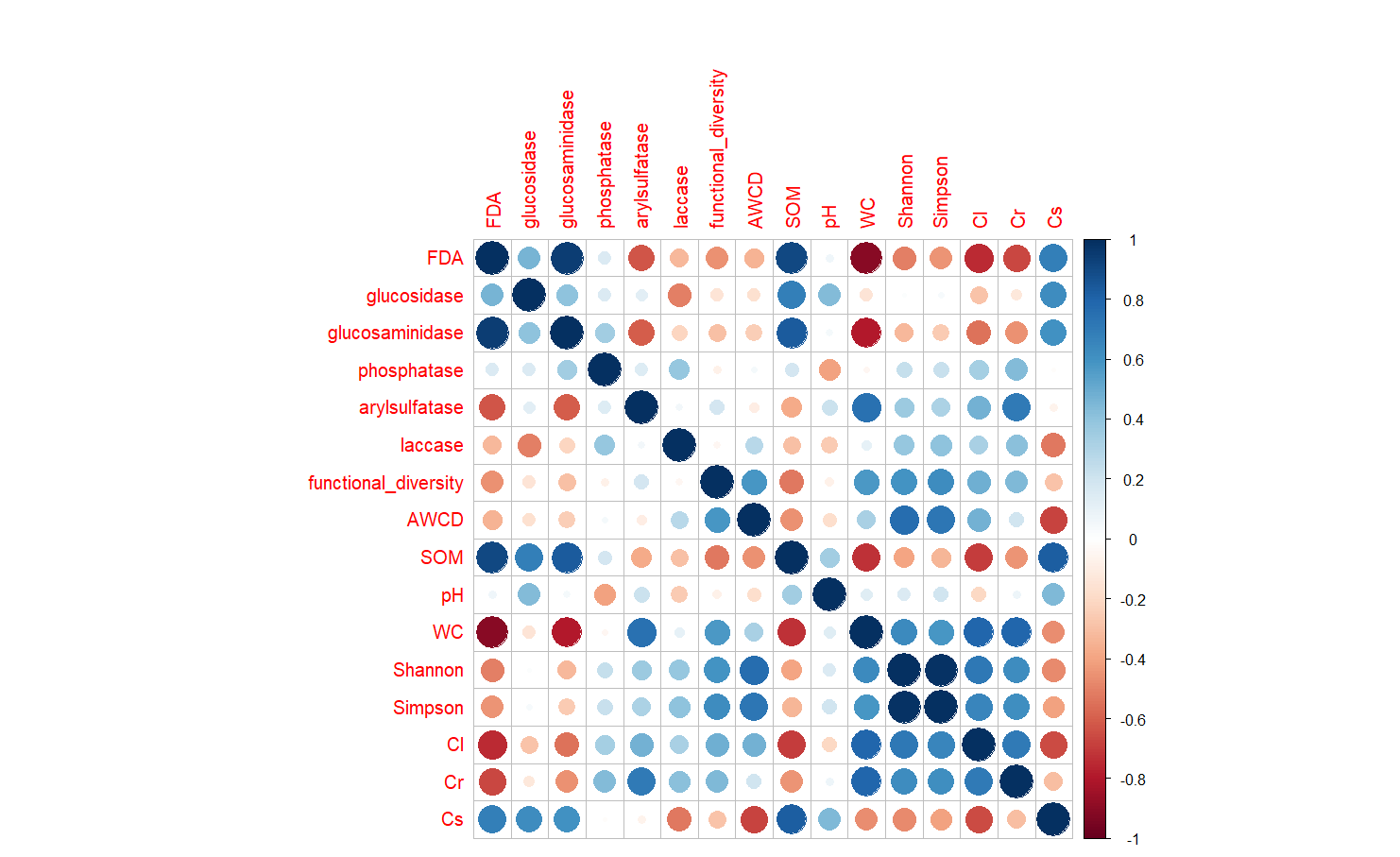


R Core Team, 2021. R: A language and environment for statistical computing. (Version 4.1.2). doi:R Foundation for Statistical Computing, Vienna, Austria. ISBN 3-900051-07-0, URL http://www.R-project.org.
